# Targeting Bcl-xL with Navitoclax Effectively Eliminates Senescent Tumor Cells That Appear Following CEP-1347-Induced Differentiation of Glioma Stem Cells

**DOI:** 10.3390/ijms26146984

**Published:** 2025-07-20

**Authors:** Senri Takenouchi, Yasufumi Ito, Kazuki Nakamura, Yurika Nakagawa-Saito, Yuta Mitobe, Keita Togashi, Shuhei Suzuki, Asuka Sugai, Yukihiko Sonoda, Chifumi Kitanaka, Masashi Okada

**Affiliations:** 1 Department of Molecular Cancer Science, Yamagata University School of Medicine, 2-2-2 Iida-Nishi, Yamagata 990-9585, Japan; 2 Department of Obstetrics and Gynecology, Yamagata University School of Medicine, 2-2-2 Iida-Nishi, Yamagata 990-9585, Japan; 3 Department of Neurosurgery, Yamagata University School of Medicine, 2-2-2 Iida-Nishi, Yamagata 990-9585, Japan; 4 Department of Ophthalmology and Visual Sciences, Yamagata University School of Medicine, 2-2-2 Iida-Nishi, Yamagata 990-9585, Japan; 5 Department of Clinical Oncology, Yamagata Prefectural Shinjo Hospital, 720-1 Kanazawa, Shinjo 996-8585, Japan; 6 Research Institute for Promotion of Medical Sciences, Yamagata University Faculty of Medicine, Yamagata 990-9585, Japan

**Keywords:** tumor-initiating cells, stem cell capacity, glioblastoma multiforme, drug repositioning

## Abstract

Cellular senescence is a state of the durable cell cycle arrest of dysfunctional cells, which has been associated with the promotion of tumor cell reprogramming into a stem cell state. We previously reported that the mixed lineage kinase (MLK) inhibitor CEP-1347 promotes the differentiation of glioma stem cells (GSCs)—key contributors to glioblastoma recurrence and therapy resistance—into non-stem tumor cells. However, we also noted that CEP-1347–treated GSCs exhibited a morphological change suggestive of senescence. Therefore, we herein investigated whether CEP-1347 induces senescence in GSCs and, consequently, if senescent GSCs may be eliminated using senolytics. Cell death induced by CEP-1347 in combination with senolytic agents or with the knockdown of anti-apoptotic *BCL2* family genes, as well as the effects of CEP-1347 on the expression of senescence markers and anti-apoptotic Bcl-2 family proteins, were examined. The results obtained showed that CEP-1347 induced senescence in GSCs accompanied by the increased expression of Bcl-xL. Among the panel of senolytic agents tested, navitoclax, a BH3 mimetic, efficiently induced cell death in GSCs when combined with CEP-1347 at concentrations clinically achievable in the brain. The knockdown of Bcl-xL resulted in more pronounced GSC death in combination with CEP-1347 than that of Bcl-2. These results suggest that combining CEP-1347 with the targeting of Bcl-xL, the expression of which increases with CEP-1347-induced senescence, is a rational approach to ensure the elimination of GSCs, thereby improving the outcomes of glioblastoma treatment.

## 1. Introduction

Glioblastoma is the most common primary malignant brain tumor and has the poorest prognosis of all primary malignant brain tumors, with an overall survival of two years or less and a five-year survival rate of 10% or lower [1,2]. Glioma stem cells (GSCs), a subtype of cancer stem cells (CSCs) in glioblastoma, are considered to play a critical role in tumor recurrence and treatment resistance, which contributes to the poor prognosis of this disease [3,4]. Accordingly, the elimination of GSCs from tumors is a promising therapeutic approach to prevent post-treatment recurrence, improve long-term outcomes, and ultimately achieve a cure.

Cellular senescence is defined as prolonged and stable cell cycle arrest that is distinct from quiescence or terminal differentiation [5,6]. Senescence may be triggered not only by organismal aging but also by various cellular insults, including oncogene activation, oxidative stress, genotoxic stress, and mitochondrial dysfunction [5,6]. In the context of cancer therapy, cellular senescence induced by radiation or chemotherapy is referred to as therapy-induced senescence [7,8]. Previous studies on the relationship between senescence and CSCs have shown that the senescence-associated secretory phenotype (SASP), whereby a collection of cytokines and other soluble factors specifically expressed by senescent cells are secreted in a paracrine manner, may promote tumorigenesis and CSC phenotypes. Furthermore, therapy-induced senescent cells are more likely to undergo reprogramming into stem-like phenotypes, and senescence itself may be a reversible process [9,10,11,12]. Therefore, the elimination of therapy-induced senescent cells within tumors using senescence-targeting agents, such as senolytics, has potential as an effective strategy to suppress the maintenance and re-emergence of CSC populations, thereby preventing post-treatment recurrence.

We previously reported that CEP-1347, the MLK inhibitor with established safety data in humans [13], induced the differentiation and suppressed self-renewal and tumorigenic potential of CSCs including GSCs, which reduced GSCs within tumors [14]. While CEP-1347 is an inducer of differentiation, we herein demonstrated for the first time that it also caused senescence in GSCs. We also investigated whether these senescent cells could be efficiently eliminated using senolytics, including navitoclax.

## 2. Results

### 2.1. CEP-1347 Induces a Senescent-like Phenotype in GSCs

**Figure 1:**
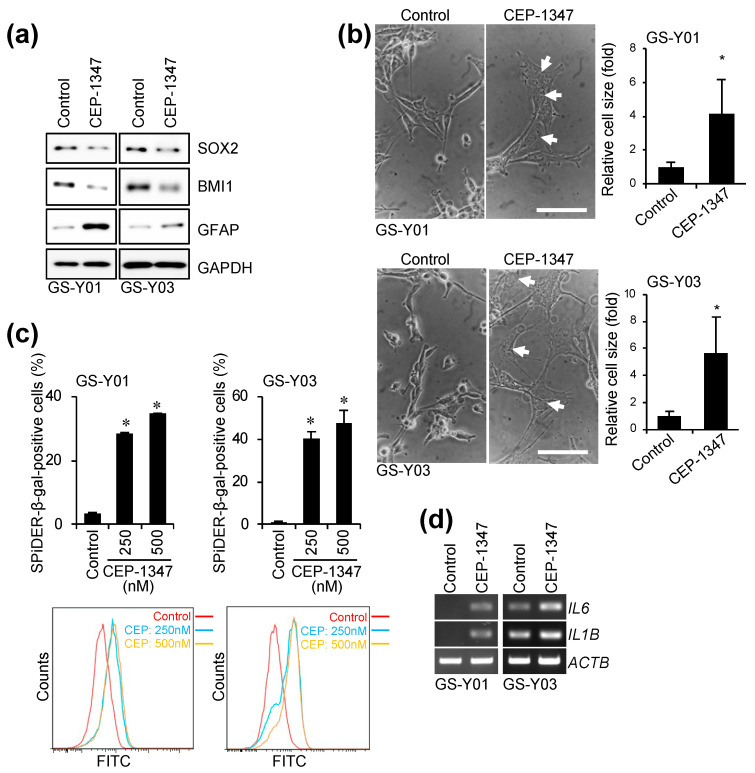
CEP-1347 induces senescence in GSCs. (**a**) GSCs (GS-Y01 and GS-Y03) treated with 250 nM CEP-1347 for three days were subjected to a Western blot analysis. (**b**) Cells were treated as in (**a**), and the area occupied by the cells was measured (right graphs). Representative phase contrast images are shown in the left panels, with multinucleated cells indicated by arrows. (**c**) Cells treated with CEP-1347 at the indicated concentrations for three days were stained with SPiDER-β-gal and analyzed by flow cytometry. The percentage of SPiDER-β-gal-positive cells (upper graphs) and representative flow cytometric histograms (lower panels) are shown. (**d**) Cells treated as in (**a**) were subjected to an RT-PCR analysis. * *p* < 0.05 vs. control. Bars: 100 µm.

We previously reported that CEP-1347, a compound with established safety data in humans, suppressed CSC-associated phenotypes and induced differentiation [14] (Figure 1a). CEP-1347 did not induce obvious cell death in GSCs during this process, but affected their morphology (Figure 1b). Specifically, GSCs became enlarged and flattened and exhibited multinucleation, suggesting that CEP-1347 induced a senescent-like phenotype in GSCs (Figure 1b). We then investigated whether CEP-1347 increased the expression of markers specific for cellular senescence in GSCs. As expected, the CEP-1347 treatment resulted in a significant increase in the number of cells that were positive for senescence-associated β-galactosidase (SA-β-gal) and up-regulated the expression of SASP factors (Figure 1c,d). These results indicate that CEP-1347 not only induced the differentiation of GSCs, but also triggered cellular senescence.

### 2.2. The Combination of CEP-1347 and Senolytics Potently Induces Cell Death in GSCs

**Figure 2:**
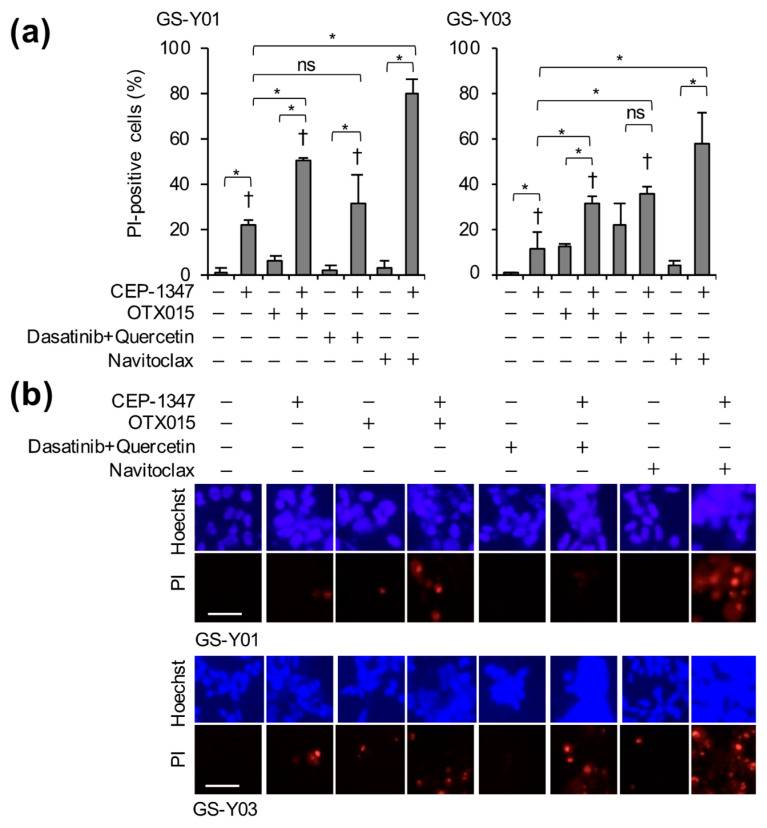
The combination of CEP-1347 and senolytics potently induces cell death in GSCs. GSCs (GS-Y01 and GS-Y03) treated as indicated with 250 nM CEP-1347, 2 μM OTX015, 100 nM dasatinib, 10 μM quercetin, and/or 500 nM navitoclax for three days were subjected to the PI uptake assay. The percentage of PI-positive cells (**a**) and representative images (**b**) are shown. * *p* < 0.05, † *p* < 0.05 vs. cells treated with 250 nM CEP-1347 in combination with 500 nM navitoclax. ns: not significant. Bars: 50 μm.

Therapy-induced senescent cells have been reported to promote stemness in non-CSCs within the tumor population via paracrine signaling, and may themselves be reprogrammed into stem-like phenotypes [9,10,11,12]. Therefore, to enhance the recurrence-suppressing effect of the CEP-1347 treatment and ultimately achieve a cure, we hypothesized that it may be necessary to eliminate CEP-1347-induced senescent cells. To test this, we examined the effects of combining CEP-1347 with several senolytics that act via different mechanisms and have known safety profiles in humans: the BET bromodomain inhibitor OTX015 [15], the combination of the tyrosine kinase inhibitor dasatinib and the natural flavonoid quercetin [16], and the Bcl-2 family inhibitor navitoclax (ABT-263) [17]. We found that the combination of CEP-1347 with OTX015 or navitoclax promoted cell death significantly more than each agent alone (Figure 2a,b). Navitoclax in combination with CEP-1347 was the most potent inducer of cell death (Figure 2a,b). These results suggest that cells treated with CEP-1347 were efficiently eliminated using senolytic agents.

### 2.3. The Combination of CEP-1347 and Bcl-xL Inhibition Potently Induces Apoptosis in GSCs

**Figure 3:**
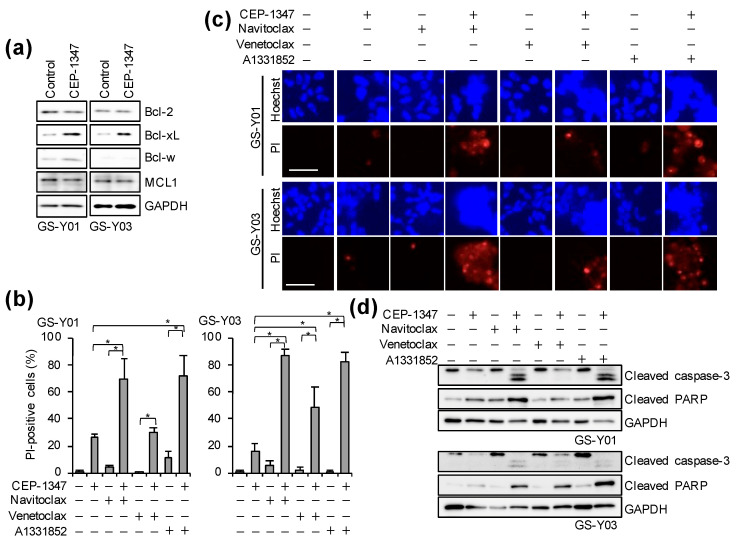
The combination of CEP-1347 and Bcl-xL targeting BH3 mimetics potently induces apoptosis in GSCs. (**a**) GSCs (GS-Y01 and GS-Y03) treated with 250 nM CEP-1347 for three days were subjected to a Western blot analysis. (**b**,**c**) GSCs treated as indicated with 250 nM CEP-1347, 500 nM navitoclax, 500 nM venetoclax, and/or 125 nM A-1331852 for three days were subjected to the PI uptake assay. The percentage of PI-positive cells (**b**) and representative images (**c**) are shown. (**d**) GSCs were treated as described in (**b**) and subjected to a Western blot analysis. * *p* < 0.05, Bars; 50 μm.

**Figure 4:**
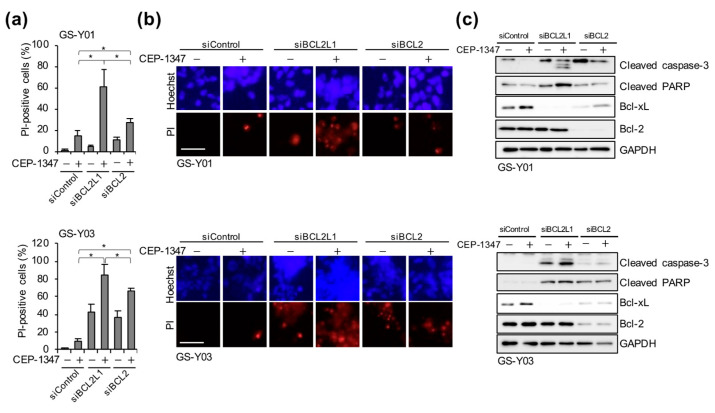
The combination of CEP-1347 and Bcl-xL depletion potently induces apoptosis in GSCs. GSCs (GS-Y01 and GS-Y03) were transiently transfected with siRNA against *BCL2* or *BCL2L1* (encoding Bcl-xL) or with control siRNA (siControl). One day after transfection, cells were treated with 250 nM CEP-1347 for three days and then subjected to the PI uptake assay (**a**,**b**) or a Western blot analysis (**c**). * *p* < 0.05, Bars: 50 μm.

As described above, the CEP-1347 treatment induced cellular senescence in GSCs, and its combination with navitoclax strongly induced cell death. Navitoclax has been shown to induce apoptosis by inhibiting anti-apoptotic Bcl-2 family members, including Bcl-2, Bcl-xL, and Bcl-w [17]. These anti-apoptotic Bcl-2 family proteins were previously shown to be up-regulated in senescent cells and contributed to their resistance to apoptosis [18,19]. Therefore, we attempted to identify which of these proteins served as the key targets for the elimination of CEP-1347–treated GSCs. We initially examined changes in the expression levels of these proteins in GSCs following the CEP-1347 treatment. While the expression of Bcl-2 and Mcl-1 did not markedly change, Bcl-w expression slightly increased and Bcl-xL expression was up-regulated (Figure 3a). We then investigated which anti-apoptotic Bcl-2 family member was the most effectively targeted for the elimination of CEP-1347–treated GSCs by using BH3 mimetics with distinct selectivity profiles: navitoclax (an inhibitor of Bcl-2, Bcl-xL, and Bcl-w), venetoclax (a Bcl-2–selective inhibitor), and A-1331852 (a Bcl-xL–selective inhibitor) [17]. Under concentrations of BH3 mimetics that showed minimal toxicity in normal fibroblasts (IMR90) (Supplemental Figure S1), we co–treated GSCs with each BH3 mimetic and CEP-1347. The combination of CEP-1347 with navitoclax or A-1331852, both of which inhibit Bcl-xL, resulted in a significantly higher percentage of dead cells than monotherapy and clearly activated caspase-3, the effector caspase, suggesting that these combinations strongly induced apoptosis (Figure 3b–d). In contrast, when combined with CEP-1347, the induction of cell death and caspase-3 activation by venetoclax, a Bcl-2–selective inhibitor, were significantly weaker than those by other BH3 mimetics that inhibit Bcl-xL (Figure 3b–d). These results suggest that Bcl-xL, which was up-regulated in response to the CEP-1347 treatment, served as a key target for BH3 mimetic–mediated senolytic apoptosis in CEP-1347–treated GSCs. Since the pharmacological inhibition of Bcl-xL was shown to be effective for eliminating CEP-1347–treated GSCs, we next investigated the molecular mechanisms underlying this combined effect using a genetic approach. Cells were transiently transfected with siRNAs targeting *BCL2* or *BCL2L1* (encoding Bcl-xL) to deplete the respective proteins, and were subsequently treated with CEP-1347. While the depletion of Bcl-2 combined with the CEP-1347 treatment led to a significantly higher percentage of dead cells than monotherapy, the combination of Bcl-xL depletion and the CEP-1347 treatment more strongly induced cell death, along with the prominent activation of caspase-3 (Figure 4). These results were consistent with the effects observed in pharmacological inhibition experiments in which CEP-1347 was combined with Bcl-2 or Bcl-xL inhibitors (Figure 3b–d). Collectively, these results demonstrate that the combination of Bcl-xL inhibition and the CEP-1347 treatment effectively eliminated senescent cells induced by CEP-1347.

### 2.4. Navitoclax Exhibits Potent GSC-Killing Activity in Combination with CEP-1347 at Clinically Achievable Concentrations for the Brain

**Figure 5:**
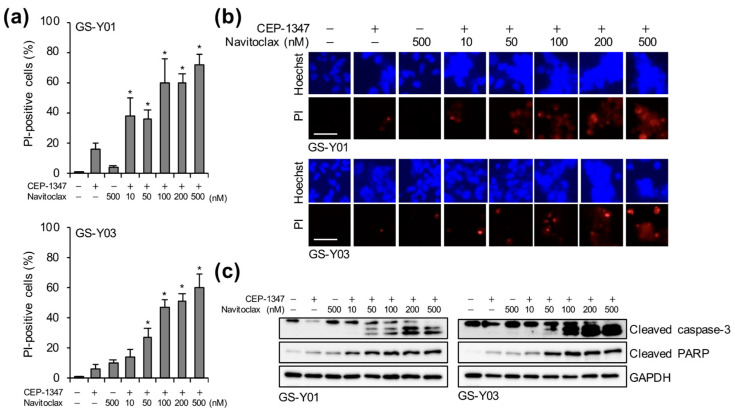
Navitoclax exhibits potent GSC-killing activity in combination with CEP-1347 at concentrations clinically achievable in the brain. GSCs (GS-Y01 and GS-Y03) treated with 250 nM CEP-1347 and/or navitoclax at the indicated concentrations for three days were subjected to the PI uptake assay. The percentage of PI-positive cells (**a**) and representative images (**b**) are shown. (**c**) GSCs were treated as described in (**a**) and subjected to a Western blot analysis. * *p* < 0.05 vs. cells treated with CEP-1347 alone. Bars: 50 μm.

In glioblastoma, the integrity of the blood–brain barrier (BBB) is known to be disrupted, at least within regions that are enhanced on contrast-enhanced T1-weighted MRI [20,21]. However, it is also clinically evident that some regions retain an intact BBB, thereby limiting drug penetration and contributing to therapeutic resistance [22,23]. A detailed pharmacokinetic study on navitoclax recently demonstrated that at a safely administrable dose, the drug reached a brain concentration of approximately 41 nM [24]. Therefore, we investigated the concentration at which navitoclax exerted cytotoxic effects on GSCs in combination with CEP-1347. Even at 50 nM, a concentration close to the reported level of 41 nM, navitoclax when co-administered with CEP-1347 resulted in a significantly higher percentage of dead cells and the stronger activation of caspase-3 than either agent alone (Figure 5). These results strongly support the potential of CEP-1347 plus navitoclax combination therapy as a realistic and effective strategy for targeting and eliminating GSCs in the brain parenchyma.

## 3. Discussion

This is the first study to demonstrate that CEP-1347, a drug we previously identified as an anti-GSC agent, induced cellular senescence in GSCs. Therapy-induced senescent cells have been reported to promote stem-like phenotypes in surrounding non-stem tumor cells via paracrine signaling and may themselves be reversibly reprogrammed into stem-like cells [9,10,11,12,25]. Moreover, senescence and stemness are both regulated by overlapping signaling pathways involving p53, p21, and p16, suggesting that senescence triggered by the activation of these pathways contributes to epigenetic reprogramming and the re-emergence of stem-like properties [9,10,11]. Therefore, even though CEP-1347 promotes the differentiation and loss of GSC-associated phenotypes, thereby suppressing the tumor-initiating capacity in the long term [14], the elimination of senescent cells induced by CEP-1347 may be of practical importance as a more robust strategy for preventing recurrence from GSC-targeted therapy and possibly achieving a cure. The present results clearly showed that senolytic agents, particularly BH3 mimetics with Bcl-xL inhibitory activity, effectively eliminated CEP-1347–treated GSCs. Interestingly, while this manuscript was in preparation, BMP4, a well-known inducer of GSC differentiation, was shown to promote senescence in glioma-initiating cells and also sensitized them to navitoclax [26], suggesting that the promotion of senescence coupled with increased sensitivity to navitoclax is not unique to CEP-1347, but may be shared, albeit not necessarily, by inducers of GSC differentiation. Therefore, the present results in concert with these findings lend support to the idea that the addition of a senolytic agent, particularly navitoclax, has potential as a therapeutic option to more effectively eliminate GSCs with inducers of GSC differentiation.

Various signaling pathways contribute to the regulation of cellular senescence, among which the p53/p21^cip1/waf1^ and p16^INK4a^/Rb pathways play central roles and have been extensively studied [27,28]. We previously reported that CEP-1347 induced the expression and activation of the p53 protein in cell lines expressing wild-type p53, including those derived from retinoblastoma [29], glioma [30], and meningioma [31]. The BMP4-induced senescence of glioma-initiating cells was accompanied by the increased expression of p21 and was also dependent on p21 expression [26]. However, it remains unclear whether CEP-1347 activated the p53 pathway in the GSC lines used in the present study, which express wild-type p53, and also if CEP-1347-induced senescence requires p21 expression. Our preliminary experiments showed that while the CEP-1347 treatment of GSCs increased the expression levels of both p53 and p21, the transient depletion of p21 only partially suppressed CEP-1347-induced, suggesting that although the p53–p21 axis is involved, other pathways may also play a major role in CEP-1347–induced senescence. In contrast to the lower frequency of TP53 alterations (27.9%) in glioblastoma, the deletion of the CDKN2A gene, which codes for p16^INK4a^ and p14^ARF^, was observed in a higher percentage of cases (57.8%), with mutations affecting Rb function being reported in up to 78.9% of cases [32]. Consistent with this finding, p16^INK4a^ expression was below the detection limit in all GSC lines used in the present study, regardless of the CEP-1347 treatment; therefore, the involvement of the p16–Rb pathway in CEP-1347-induced senescence currently remains unclear. Apparently, further studies are needed to investigate other mechanisms underlying CEP-1347–induced senescence, including p16^INK4a^-independent Rb regulation or alternative signaling pathways. In this regard, Aurora kinases may be of interest given the known inhibitory effect of CEP-1347 on Aurora kinases [33], since MLN8054, a selective Aurora A inhibitor, has been shown to induce increased levels of both p53 and p21, cellular and nuclear enlargement, and SA-β-gal staining [34]. The involvement of JNK in CEP-1347-induced senescence, which acts downstream of MLKs targeted by CEP-1347, is also a possibility, since JNK inhibition reportedly resulted in the induction of senescence-like phenotype characterized by mitotic inhibition, multinucleation, and delayed apoptosis [35].

In the present study, we investigated the senolytic agents OTX015, dasatinib combined with quercetin, and navitoclax as potential therapeutics for eliminating CEP-1347–treated GSCs. The safety of these compounds has been evaluated in clinical trials on human subjects. The maximum plasma concentrations (Cmax) observed at the maximum tolerated doses in phase I dose-escalation studies were 1529 µg/L (3.11 μM) for OTX015 [36], 127.10 ng/mL (0.260 μM) for dasatinib [37], and 3.56 µg/mL (3.65 μM) for navitoclax [38]. The concentrations used in our combination experiments with CEP-1347 were 2 μM for OTX015 and 100 nM for dasatinib, which were approximately two-thirds and two-fifths of their respective clinical Cmax values, and 500 nM for navitoclax, which was approximately one-seventh of its reported Cmax. Since navitoclax, at a concentration below its clinical Cmax, more potently induced cell death when combined with CEP-1347 than the other two senolytics, the present results suggest that navitoclax has the greatest therapeutic potential for eliminating CEP-1347–induced senescent GSCs. Furthermore, navitoclax [24] and A-1331852 [39] have both been reported to possess BBB permeability. We herein demonstrated that even at low concentrations achievable intracerebrally by the systemic administration of navitoclax [24], its combination with CEP-1347 strongly induced cell death in GSCs. Therefore, the combination of CEP-1347 and navitoclax may effectively eliminate GSCs in glioblastomas developing within the brain in vivo. However, the intracerebral concentration of A-1331852 following its systemic administration has not yet been clarified, and the present study did not investigate whether the combination of CEP-1347 and A-1331852 exerted similar GSC-killing effects at physiologically relevant brain concentrations. Importantly, since Bcl-xL plays a critical role in platelet survival, its pharmacological inhibition has been shown to cause thrombocytopenia [24,40]. Therefore, future research needs to focus on evaluating both the therapeutic efficacy and tolerability of these combination treatments using xenograft models, particularly orthotopic brain tumor models.

Collectively, the present results demonstrate that CEP-1347, which induces the differentiation of GSCs, also rendered them eliminable through the induction of cell death when combined with senolytics, particularly Bcl-xL inhibitors. These combination treatments may serve as a novel therapeutic strategy for glioblastoma by suppressing de novo tumor initiation through the enforced differentiation of GSCs into non–self-renewing cells while also eliminating senescent GSC-derived cells induced by CEP-1347, thereby preventing the re-emergence of GSCs in the long term.

## 4. Materials and Methods

### 4.1. Antibodies and Reagents

Antibodies against cleaved caspase-3 (#9661), cleaved PARP (#9541), GFAP (#3670), p21 (#2947), Bcl-2 (#15071), Bcl-xL (#2764), and GAPDH (#5174) were purchased from Cell Signaling Technology, Inc. (Beverly, MA, USA). An antibody against Bmi-1 (05-637) was obtained from Merck KGaA (Darmstadt, Germany). Antibodies against p53 (sc-126) and Mcl-1 (sc-20679) were from Santa Cruz Biotechnology, Inc. (Dallas, TX, USA). An antibody against SOX2 (MAB2018) was supplied by R&D Systems, Inc. (Minneapolis, MN, USA). An antibody against BCL2L2/BCL-w (16026-1-AP) was purchased from ProteinTech Group, Inc. (Rosemont, IL, USA). Horseradish peroxidase (HRP)-conjugated rabbit and mouse secondary antibodies were from Jackson ImmunoResearch, Inc. (West Grove, PA, USA). CEP-1347 was purchased from TOCRIS Bioscience (Bristol, UK). OTX015, dasatinib, quercetin, and A-1331852 were from Cayman Chemicals (Ann Arbor, MI, USA). Navitoclax (ABT-263) was purchased from Chemscene (Monmouth Junction, NJ, USA). Venetoclax (ABT-199) was purchased from LC Laboratories (Woburn, MA, USA).

### 4.2. Cell Culture

The isolation, establishment, and characterization of the stem-like properties of patient-derived GSCs (GS-Y01 and GS-Y03) were conducted as previously described [41,42]. GSCs were maintained under previously reported monolayer stem cell culture conditions [43,44]. The differentiation of GSCs was induced by culturing cells in DMEM/F12 medium supplemented with 10% fetal bovine serum (FBS, Thermo Fisher Scientific, Inc., Waltham, MA, USA), 100 units/mL of penicillin, and 100 μg/mL of streptomycin for 7–14 days [43,44]. IMR90, a human normal fetal lung fibroblast cell line, was obtained from the American Type Culture Collection (Manassas, VA, USA) and maintained in DMEM supplemented with 10% FBS. All IMR90 experiments were performed using cells with a low passage number (<8). Cell area was measured using ImageJ software (version 1.53k).

### 4.3. Propidium Iodide (PI) Uptake Assay

The PI uptake assay was performed to assess the percentage of dead cells as previously described [43,45]. Fluorescent images were obtained using a fluorescence microscope (CKX53; EVIDENT, Tokyo, Japan) equipped with an iPhone 7 (Apple, Cupertino, CA, USA) and scored.

### 4.4. Western Blot Analysis

A Western blot analysis was conducted as previously described [43,45]. Cells were harvested, washed with ice-cold phosphate-buffered saline (PBS), and then lysed in RIPA buffer (10 mM Tris/HCl (pH 7.4), 0.1% sodium dodecyl sulfate (SDS), 0.1% sodium deoxycholate, 1% Nonidet P-40, 150 mM NaCl, 1 mM EDTA, 1.5 mM sodium orthovanadate, 10 mM sodium fluoride, 10 mM sodium pyrophosphate, and protease inhibitor cocktail set III (FUJIFILM Wako Pure Chemical Corporation, Osaka, Japan)). The lysate was immediately mixed with the same volume of 2 × Laemmli buffer (125 mM Tris/HCl (pH 6.8), 4% SDS, and 10% glycerol) and boiled at 95 °C for 10 min. Samples with protein concentrations measured using a BCA protein assay kit (Thermo Fisher Scientific) were separated by SDS/polyacrylamide gel electrophoresis and transferred to polyvinylidene difluoride membranes. The membranes were reacted with primary antibodies followed by appropriate HRP-conjugated secondary antibodies as recommended by the manufacturer of each antibody, and then detected using Immobilon Western Chemiluminescent HRP Substrate (Merck). To reprobe immunoblots, antibodies were stripped from the probed membranes using stripping buffer (2% SDS, 100 mM β-mercaptoethanol, and 62.5 mM Tris-HCl (pH 6.8)). After stripping, the membranes were washed with Tris-buffered saline with Tween 20, blocked with skim milk, and reprobed with appropriate antibodies. Immunoreactive bands were detected by a ChemiDoc Touch device (Bio-Rad Laboratories, Inc., Hercules, CA, USA).

### 4.5. Reverse Transcription (RT)-PCR Analysis

An RT-PCR analysis was performed as previously described [43]. Total RNA was extracted from cells using Trizol (Thermo Fisher Scientific), and RNA was reverse transcribed using the PrimeScript RT reagent kit (Takara Bio Inc., Shiga, Japan) according to the manufacturer’s protocol. The target genes were amplified with Quick Taq HS DyeMix (Toyobo Co., Ltd., Osaka, Japan) using the primer sets listed below: *IL6* (Fw: 5′-AAAGAGGCACTGGCAGAAAAC-3′, Rv: 5′-AGCTCTGGCTTGTTCCTCAC-3′), *IL1B* (Fw: 5′-AACAGGCTGCTCTGGGATTC-3′, Rv: 5′-AGTCATCCTCATTGCCACTGT-3′), and *ACTB* (Fw: 5′-CCCATGCCATCCTGCGTCTG-3′, Rv: 5′-CGTCATACTCCTGCTTGCTG-3′).

### 4.6. Fluorescence-Activated Cell Sorting (FACS) Analysis

Cells were stained with 1 μM SPiDER-β-Gal (SG02, DOJINDO LABORATORIES, Kumamoto, Japan) in Hanks’ Balanced Salt Solution (HBSS) at 37 °C for 15 min. After being washed with HBSS twice, cells were fixed in 4% paraformaldehyde at RT for 15 min, resuspended in PBS, and examined using FACSCanto II (BD Biosciences, Franklin Lakes, NJ, USA). The data obtained were analyzed using FlowJo software, version 7.6.5 (Tree Star Inc., Ashland, OR, USA).

### 4.7. Gene Silencing by siRNA

AllStars Negative Control siRNA (QIAGEN, Venlo, The Netherlands) or siRNAs against *BCL2* (HSS100956) and *BCL2L1* (HSS141361) (Thermo Fisher Scientific) were transfected using Lipofectamine RNAiMAX (Thermo Fisher Scientific) in accordance with the manufacturer’s instructions.

### 4.8. Data Reproducibility and Statistical Analysis

PI uptake assays, the Western blot analysis, RT-PCR, and the FACS analysis were repeated at least twice with similar results, and one set of representative data is presented. Data analyses were performed using the software Microsoft Excel (Version 2402 or Ver16.66.1, Redmond, WA, USA). Results are expressed as the mean and standard deviation, and the significance of differences was assessed using Student’s two-tailed *t*-test for comparisons of two groups. *p* values < 0.05 were considered to be significant and are indicated with asterisks in the figures.

## Data Availability

All data are contained in this article and there are no repository data.

## Supplementary Materials

The following supporting information may be downloaded at: https://www.mdpi.com/article/10.3390/ijms26146984/s1.
