# Supplementary material for: Targeting Bcl-xL with Navitoclax Effectively Eliminates Senescent Tumor Cells That Appear Following CEP-1347-Induced Differentiation of Glioma Stem Cells
Source: Int J Mol Sci. 2025 Jul 20;26(14):6984. doi: 10.3390/ijms26146984 (PMC12294909; doi:10.3390/ijms26146984)
Supplement: Supplementary file 1 [file ijms-26-06984-s001.zip › ijms-3720153-supplementary.pdf]

Supplemental Figure S1

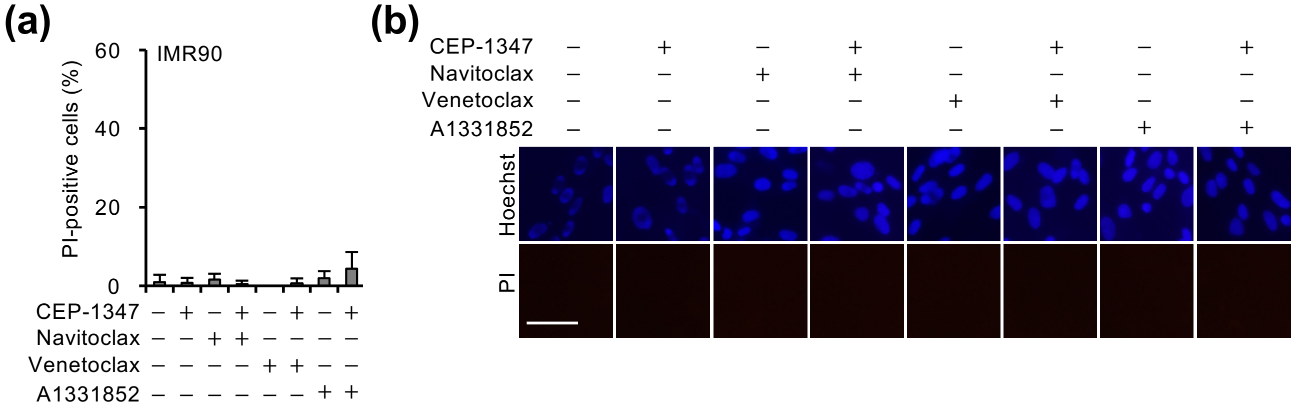

Supplemental Figure S1. Effects of the combination of CEP-1347 and BH3 mimetics on IMR90 cells.

IMR90 cells treated as indicated with 250 nM CEP-1347, 500 nM navitoclax, 500 nM venetoclax, and/or 125 nM A-1331852 for three days were subjected to the PI uptake assay. The percentage of dead cells (a) and representative images (b) are shown. Bar: 50 µm.
